# Turbo-RIP: A Protocol for TurboID-based RNA Immunopurification to Map RNA Landscapes in Plant Biomolecular Condensates

**DOI:** 10.21769/BioProtoc.5587

**Published:** 2026-02-05

**Authors:** Zhi Zhang, Yanting Xu, Hanxiang Liu, Chen Liu, Panagiotis Nikolaou Moschou

**Affiliations:** 1 State Key Laboratory of Biocontrol, Guangdong Key Laboratory of Plant Stress Biology, Innovation center for evolutionary synthetic biology, School of Life Sciences, Sun Yat-Sen University, Guangzhou, China; 2 Department of Biology, University of Crete, Heraklion, Greece; 3 Institute of Molecular Biology and Biotechnology, Foundation for Research and Technology-Hellas, Heraklion, Greece; 4 Department of Molecular Sciences, Uppsala BioCenter, Swedish University of Agricultural Sciences and Linnean Center for Plant Biology, Uppsala, Sweden

**Keywords:** TurboID, Proximity biotinylation, RNA immunopurification, Biomolecular condensates, Liquid–liquid phase separation, Plant stress responses

## Abstract

Biomolecular condensates organize cellular processes through liquid–liquid phase separation, creating membrane-less compartments enriched in specific proteins and RNAs. Understanding their RNA composition is essential for elucidating plant stress responses, yet capturing these transiently associated RNAs remains technically challenging. We present Turbo-RIP (TurboID-based proximity labeling with RNA immunopurification), a comprehensive protocol for identifying condensate-associated RNAs in plants. Turbo-RIP employs the biotin ligase TurboID to label proximal proteins at 22 °C, followed by formaldehyde crosslinking and streptavidin-based capture of protein–RNA complexes. We provide detailed procedures for three cloning strategies, transformation of *Nicotiana benthamiana* and *Arabidopsis thaliana*, validation of TurboID activity, and RNA recovery. The protocol successfully captured processing body–associated RNAs with minimal background. Turbo-RIP enables systematic mapping of RNA populations within plant condensates under diverse conditions. The protocol requires 3–5 days from sample preparation to RNA isolation, with construct validation taking 2–4 weeks. All procedures use standard laboratory equipment, making Turbo-RIP accessible for plant molecular biology laboratories.

Key features

• Apply TurboID-based proximity labeling specifically for RNA capture in plant condensates.

• Optimize experimental conditions for different plant species and condensate types.

• Implement quality control measures and data analysis pipelines.

## Graphical overview


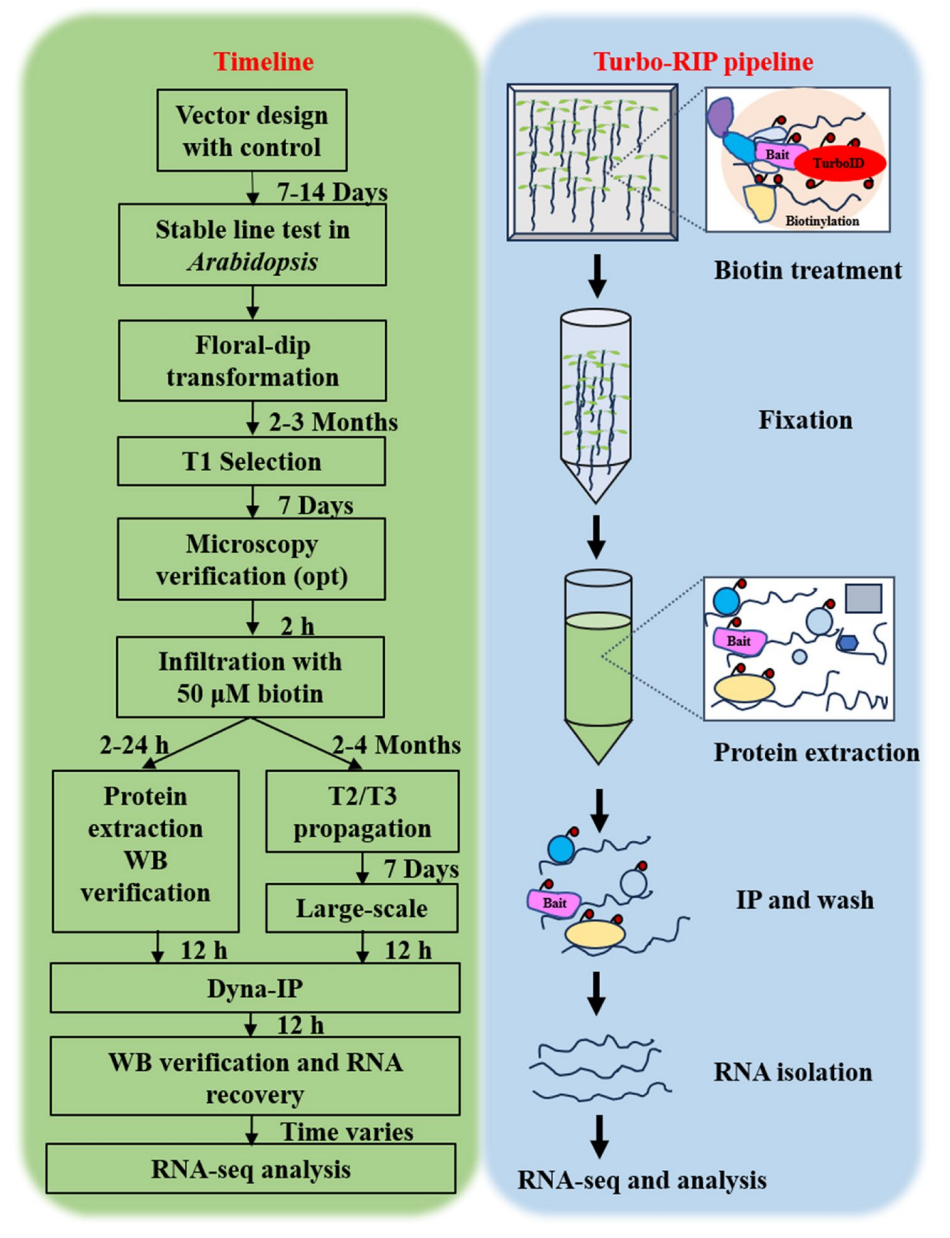


**Turbo-RIP.** T, transformation. Opt, optional. WB, western blot. IP, immunopurification. Dyna-IP, Dynabeads^TM^ MyOne Streptavidin C1 immunopurification.

## Background

Biomolecular condensates are dynamic assemblies formed through liquid–liquid phase separation (LLPS), creating specialized cellular microenvironments without membrane boundaries [1,2]. These structures compartmentalize specific proteins, RNAs, and metabolites, enabling rapid cellular responses to environmental changes. In plants, condensates regulate diverse processes including RNA processing, stress signaling, and metabolic channeling [3,4].

Traditional approaches for studying condensate composition face significant limitations. Crosslinking and immunoprecipitation (CLIP) methods often require highly specific antibodies and fail to capture the transient interactions characteristic of condensates [5]. The tandem RNA isolation procedure (TRIP) requires stable expression of RNA and binding proteins, which can be time-consuming. Hence, using TRIP, weak or highly dynamic interactions may be lost during a stringent purification [6,7]. Overall, the dynamic exchange of components and weak multivalent interactions that drive phase separation make compositional analysis particularly challenging.

Here, we present Turbo-RIP (TurboID-based proximity labeling with RNA immunopurification), a robust protocol that combines proximity-dependent biotinylation with RNA capture techniques. The method leverages TurboID, an engineered *Escherichia coli* biotin ligase that overcomes the temperature limitations of earlier BioID variants, functioning efficiently at plant-compatible temperatures (22 °C) [8]. TurboID catalyzes the formation of reactive biotin-AMP, which covalently modifies lysine residues on proteins within ~10 nm radius [9]. By capturing biotinylated RNA-binding proteins and their associated RNAs, Turbo-RIP enables comprehensive mapping of condensate RNA landscapes. The overall protocol is a modification of the APEAL approach (Tandemly Coupled Affinity Purification with Proximity-dEpendent LigAtion), which we have detailed previously [10,11].

## Materials and reagents

**Biological materials**

1. Transient expression system: healthy *Nicotiana benthamiana* plants, 3–5 weeks old

2. *Arabidopsis thaliana Columbia-0* (Col-0, five-six weeks) for construction of transgenic lines: TurboID-tagged protein of interest-expressing lines (protein of interest-TurboID) and TurboID-expressing lines (control line) [8]; select 8–10 independent lines via antibiotic plates and pick two lines of each for western blot (WB) verification and further experiments [12]. Five-day-old seedlings of transgenic lines are used for biotin incubation and other tests

3. Bacterial strain NEB^®^ 10-beta Competent *Escherichia coli* (New England Biolabs, catalog number: C3019H) DH10B, genotype: *Δ(ara-leu) 7697 araD139 fhuA ΔlacX74 galK16 galE15 e14- ϕ80dlacZΔM15 recA1 relA1 endA1 nupG rpsL (StrR) rph spoT1 Δ(mrr-hsdRMS-mcrBC)*

4. Electrocompetent Agrobacterium (*Agrobacterium tumefaciens*) strain C58C1 Rif^R^ (pMP90) or GV3101 Rif^R^

**Reagents**

*Note: Reagents are kept at room temperature (RT) or other conditions according to the manufacturer's instructions. Reactions are performed according to the manufacturer's instructions unless otherwise specified.*

1. LB broth/LB agar (Duchefa Biochemie, catalog numbers: L1704 and L1706)

2. MS plant medium (Murashige and Skoog medium) (Duchefa Biochemie, catalog number: M0221)

3. Gelrite (Duchefa Biochemie, catalog number: G1101)

4. Nuclease-free water (Sigma-Aldrich, catalog number: W4502)

5. 10 mM dNTP (Thermo Fisher Scientific, catalog number: 18427088)

6. Biotin (Sigma-Aldrich, catalog number: 2031), store at 4 °C, protect from light

7. 4′-Hydroxy-3′,5′-dimethoxyacetophenone, acetyl syringone (acetosyringone) (Sigma-Aldrich, catalog number: D134406), store at RT, protect from light

8. 2-(N-morpholino) ethanesulfonic acid (MES) (Sigma-Aldrich, catalog number: 4432-31-9)

9. 37% (w/v) formaldehyde (Sigma-Aldrich, catalog number: 1.04002.1000)

10. Glycine (Sigma-Aldrich, catalog number: 1.04201.0100)

11. Tri (hydroxymethyl) aminomethane (Tris) (Sigma-Aldrich, catalog number: 1.08387)

12. Ethylenediamine tetraacetic acid disodium salt (EDTA) (Sigma-Aldrich, catalog number: 6381-92-6)

13. Sodium chloride (NaCl) (Thermo Fisher Scientific, catalog number: S271-10)

14. Magnesium chloride (MgCl_2_) (VWR, catalog number: MK5958-04)

15. Calcium chloride (CaCl_2_) (VWR, catalog number: MK5958-04)

16. Sodium dodecyl sulfate (SDS) (MilliporeSigma, catalog number: L3771-1KG)

17. Triton X-100 (MilliporeSigma, catalog number: T9284-500 mL)

18. Glycerol (Sigma‐Aldrich, catalog number: G5516)

19. Dithiothreitol (DTT) (Thermo Fisher Scientific, catalog number: BP172-5)

20. Phenylmethylsulphonyl fluoride (PMSF) (Sigma-Aldrich, catalog number: 195381)

21. Natrium azide (Na-Azide) (Merk, catalog number: S28222 917)

22. Diethyl pyrocarbonate (DEPC) (Sigma-Aldrich, catalog number: 016K3726)

23. Ethanol (EtOH) (Sigma-Aldrich, catalog number: 1.00974.2511)

24. Methanol (MeOH) (Millipore, catalog number: 34966-4 L)

25. Chloroform (Sigma-Aldrich, catalog number: 1.02444.1000)

26. TurboID plasmids and the corresponding codon-optimized sequence for plant-specific expression can be found in [8]; the original plasmids can be obtained from Addgene (https://www.addgene.org/search/catalog/plasmids/?q=turboid)

27. Phusion^TM^ High‐Fidelity DNA Polymerase & dNTP mix or equivalent proofreading polymerase (Thermo Fisher Scientific, catalog number: F530S)

28. In Fusion^®^ HD Cloning kit (Clonetech/Takara, catalog number: 638909)

29. Gateway^TM^ BP Clonase^TM^ II enzyme mix (Thermo Fisher Scientific, catalog number: 11789-020)

30. Gateway^TM^ LR Clonase^TM^ II enzyme mix (Thermo Fisher Scientific, catalog number: 11791020)

31. BsaI–HF Type IIS restriction enzyme optimized for Golden Gate Assembly (New England BioLab, catalog number: 10156743)

32. T4 DNA ligase (New England BioLab, catalog number: M0202S)

33. pENTR^TM^/D−TOPO^®^ Cloning kit, with One Shot^®^ TOP10 Chemically Competent *E. coli* (Thermo Fisher Scientific, catalog number: ab178021)

34. Primary vectors: pDONR221 (GatewayTM) and pGWB501/601 [8] or similar

35. Rifampicin (Duchefa Biochemie, catalog number: R0146.0005)

36. Kanamycin (Duchefa Biochemie, catalog number: K0126.0005)

37. Hygromycin B (Duchefa Biochemie, catalog number: H0192)

38. Spectinomycin (Duchefa Biochemie, catalog number: S0188.0005)

39. GeneJET Gel Extraction kit (Thermo Fisher Scientific, catalog number: K0692)

40. GeneJET Plasmid Miniprep kit (Thermo Fisher Scientific, catalog number: K0503)

41. Dynabeads^TM^ MyOne Streptavidin C1 (Thermo Fisher Scientific, catalog number: 65601), store at 4 °C, ≥2,800 pmol free biotin/mg beads

42. DynaMag^TM^-2 magnet (Thermo Fisher Scientific, catalog number: 112321D)

43. PD‐10 columns (Cytiva, catalog number: 17085101)

44. Protease inhibitor cocktail (Sigma‐Aldrich, catalog number: P9599)

45. RNase inhibitor (40 U/μL) (Thermo Fisher Scientific, catalog number: EO0381)

46. Proteinase K (Thermo Fisher Scientific, catalog number: 25530049, 20 mg/mL), store at -20 °C

47. Phenol-chloroform-isoamyl alcohol mixture (25:24:1) pH 4.0–5.0 (VWR, catalog number: 136112-00-0)

48. Isopropanol (2-propanol) (VWR, catalog number: BDH1133-4LP)

49. *β-*Mercaptoethanol (Sigma‐Aldrich, catalog number: 444203)

50. Sodium acetate (NaAc) (Sigma‐Aldrich, catalog number: S8750)

51. GlycoBlue (Thermo Fisher Scientific, catalog number: AM951, 15 mg/mL), nuclease and protease-free

52. Polyoxyethylene (20) sorbitan monolaurate (Tween 20) (Sigma-Aldrich, catalog number: 655204)

53. IGEPAL (CA‐630) (Sigma‐Aldrich, catalog number: MK5958-04)

54. 4 x Laemmli buffer (Bio‐Rad, catalog number: 1610747)

55. 30% acrylamide/Bis solution (29.2:0.8) (Bio-Rad, catalog number: 1610156)

56. Ammonium persulfate (APS) (Sigma-Aldrich, catalog number: A3678)

57. N,N,N,N′-Tetramethyl-ethylenediamine (TEMED) (Sigma-Aldrich, catalog number: T7024)

58. Any kD^TM^ Mini-PROTEAN^®^ TGX^TM^ Precast Protein Gels, 10-well format or similar (Bio-Rad, catalog number: 4569033)

59. Bovine serum albumin fraction V (BSA) (Sigma-Aldrich, catalog number: 9048-46-8)

60. PageRuler^TM^ Plus Prestained Protein Ladder or similar (Thermo Fisher Scientific, catalog number: 26619)

61. Polyvinylidene fluoride membrane (PVDF), 0.22 μm, 30 × 3 M (Bio‐Rad, catalog number: 1620177 or similar)

62. Phosphate-buffered saline (PBS) tablet (Sigma‐Aldrich, catalog number: P4417)

63. 3′,3″,5′,5″-tetrabromophenolsulfonphthalein (Bromophenol blue) (Sigma-Aldrich, catalog number: B5525)

64. Streptavidin-horseradish peroxidase (Strep-HRP) (Sigma-Aldrich, catalog number: GERPN1231 or similar), use in dilution of 1:25,000 in 3% BSA with 1× PBST

65. Mouse anti‐FLAG‐horseradish peroxidase (FLAG-HRP) (Sigma-Aldrich, catalog number: A8592 or similar), use in dilution 1:25,000 in 3% BSA with 1× PBST

66. Rat anti‐Tubulin (Santa Cruz Biotechnology, 1:1,000)

67. Anti‐rat [IRDye^®^ 800 CW Goat α‐Rat IgG (H + L), LI‐COR, 925‐32219, 1:10,000]

68. Enhanced chemiluminescence (ECL) HRP substrate of regular sensitivity, typically as a two-component system consisting of a stable peroxide solution and an enhanced luminol solution (Cytiva or similar)

69. ECL of high sensitivity: SuperSignal^TM^ West Femto Maximum Sensitivity Substrate (Thermo Fisher Scientific, catalog number: 34094 or similar)

70. RNase-free DNase (e.g., RQ1 from Promega); RQ1 RNase-Free DNase is provided with the following reagents: 10× RQ1 RNase-free DNase buffer, RQ1 RNase-free DNase, and RQ1 DNase stop solution

71. 100 μM oligo d(T) and/or 50 μM random hexamers and/or specific primers against known RNA

72. Qubit^®^ RNA HS Assay kit (Thermo Fisher Scientific, catalog number: Q32852)

73. NEBNext^® ^Ultra^TM^ II RNA Library Prep with sample purification beads (New England BioLab, catalog number: E7775S)

74. NEBNext Multiplex Oligos for Illumina (Dual Index Primers Set 1) (New England BioNordika BioLab, catalog number: E7600S)

75. RiboMinus^TM^ Plant kit (Thermo Fisher Scientific, catalog number: A1083808)

76. High-performance reverse transcriptase (Thermo Fisher Scientific, catalog number: 18090200); SuperScript IV is provided with the following reagents: 5× SSIV buffer, 0.1 M DTT, and SuperScript IV reverse transcriptase

77. qRT-PCR quantification master mix (e.g., Thermo Fisher Scientific, catalog number: 11813923)

78. RNaseZap^®^ RNase decontamination solution (ThermoFisher Scientific, catalog number: AM9780)

79. Agilent DNA 7500 kit (Agilent, catalog number: 5067-1506)

80. Appropriate primer set to detect the RNA/cDNA of interest as well as negative and positive controls if possible [e.g., EBF1f (5′-3′): TCAGATCTTTAGTTTTGCCGGTGA; EBF1r (5′-3′): TGCTACTTACAAGCGTAAGCCA]

**Solutions**

*Note: Prepare all solutions using ultrapure water (DNase-, RNase-, and protease-free, obtained by purifying deionized water to achieve a resistivity of 18 MΩ·cm at 25 °C) or another specified solvent, using analytical-grade reagents. Store all reagents at room temperature unless otherwise indicated. Exercise general safety precautions when handling hazardous chemicals: wear gloves, work in a fume hood when necessary, and strictly follow all regulations for waste disposal.*

1. Diethyl pyrocarbonate (DEPC) water (see Recipes)

2. 1.5 M Tris-HCl, pH 8.8 (see Recipes)

3. 0.5 M Tris-HCl, pH 6.8 (see Recipes)

4. 1 M Tris-HCl, pH 8.0 (see Recipes)

5. 1 M Tris-HCl, pH 7.5 (see Recipes)

6. LB medium (see Recipes)

7. Half-strength (1/2) MS plant medium (see Recipes)

8. 50% glycerol (see Recipes)

9. 1 M MgCl_2_ (see Recipes)

10. 1 M CaCl_2 _(see Recipes)

11. 5 M NaCl (see Recipes)

12. 0.5 M EDTA, pH 8.0 (see Recipes)

13. 1 M DTT (see Recipes)

14. 100 mM acetosyringone (see Recipes)

15. 50 mM biotin (see Recipes)

16. 0.5 M MES (see Recipes)

17. Infiltration buffer (see Recipes)

18. Kanamycin 100 mg/mL (see Recipes)

19. Spectinomycin 50 mg/mL (see Recipes)

20. Rifampicin 25 mg/mL (see Recipes)

21. 2 M glycine (see Recipes)

22. 20% (v/v) Triton X-100 (see Recipes)

23. Extraction buffer (EB) (see Recipes)

24. Wash buffer (see Recipes)

25. Dilution buffer (see Recipes)

26. Elution buffer (see Recipes)

27. 10% (w/v) SDS (see Recipes)

28. 10% (w/v) APS (see Recipes)

29. 10× SDS running buffer (see Recipes)

30. 1× SDS transfer buffer (see Recipes)

31. 10× PBS (see Recipes)

32. 3% (w/v) BSA blocking solution (see Recipes)

33. 0.1% (w/v) Ponceau staining solution (see Recipes)

34. Protease K buffer (see Recipes)

35. Homogenization buffer (see Recipes)

36. 3 M NaAc (see Recipes)

**Recipes**

*Note: We followed the manufacturer’s recommendations to adjust the pH of solutions at 4 °C. The procedure presented in this protocol is mainly performed at cold temperatures or on ice to prevent RNA or protein degradation, so the pH of solutions must be adjusted at 4 °C as the working temperature for the protocol. The volume of all solutions is adjusted with DEPC water unless specifically indicated.*

**1. DEPC water**

**Table:** 

Reagent	Final concentration	Quantity or volume
DEPC	0.1%	1 mL
Total	n/a	1,000 mL

Stir overnight and autoclave. Store at RT.

**2. 1.5 M Tris-HCl, pH 8.8**

**Table:** 

Reagent	Final concentration	Quantity or volume
Tris-HCl	1.5 M	181.7 g
Total	n/a	1,000 mL

Adjust pH with 6 N HCl, autoclave, and store at 4 °C.

**3. 0.5 M Tris-HCl, pH 6.8**

**Table:** 

Reagent	Final concentration	Quantity or volume
Tris-HCl	0.5 M	60.6 g
Total	n/a	1,000 mL

Adjust pH with 6 N HCl, autoclave, and store at 4 °C.

**4. 1 M Tris-HCl, pH 8.0**

**Table:** 

Reagent	Final concentration	Quantity or volume
Tris-HCl	1 M	121.1 g
Total	n/a	1,000 mL

Adjust pH with 6 N HCl, autoclave, and store at 4 °C.

**5. 1 M Tris-HCl, pH 7.5**

**Table:** 

Reagent	Final concentration	Quantity or volume
Tris-HCl	1 M	121.1 g
Total	n/a	1,000 mL

Adjust pH with 6 N HCl, autoclave, and store at 4 °C.

**6. LB medium**

**Table:** 

Reagent	Final concentration	Quantity or volume
Peptone	1% (w/v)	10 g
Yeast extract	0.5% (w/v)	5 g
NaCl	1% (w/v)	10 g
Agar	1.2% (w/v)	12 g
Total	n/a	1,000 mL

Autoclave and store at RT.

**7. Half-strength (1/2) MS plant medium**

**Table:** 

Reagent	Final concentration	Quantity or volume
MS	2.15 g/L	2.15 g
Sucrose	1% (w/v)	10 g
Gelrite	1% (w/v)	10 g
Total	n/a	1,000 mL

Use 1 M KOH to adjust pH to 5.8, add gelrite, autoclave, and store at 4 °C.

**8. 50% glycerol**

**Table:** 

Reagent	Final concentration	Quantity or volume
Glycerol	50% (v/v)	50 mL
Total	n/a	100 mL

Autoclave and store at RT. Alternatively, filter-sterilize using a 0.22 μm filter and store at RT.

**9. 1 M MgCl_2_**

**Table:** 

Reagent	Final concentration	Quantity or volume
MgCl_2_·6H_2_O	1 M	20.33 g
Total	n/a	100 mL

Autoclave and store at RT.

**10. 1 M CaCl_2_**

**Table:** 

Reagent	Final concentration	Quantity or volume
CaCl_2_·2H_2_O	1 M	14.702 g
Total	n/a	100 mL

Autoclave and store at RT.

**11. 5 M NaCl**

**Table:** 

Reagent	Final concentration	Quantity or volume
NaCl	5 M	29.22 g
Total	n/a	100 mL

Autoclave and store at RT.

**12. 0.5 M EDTA, pH 8.0**

**Table:** 

Reagent	Final concentration	Quantity or volume
EDTA	0.5 M	14.621 g
Total	n/a	100 mL

Use 10 N NaOH to adjust pH to 8.0.

**13. 1 M DTT**

**Table:** 

Reagent	Final concentration	Quantity or volume
DTT	1 M	1.54 g
Total	n/a	10 mL

Dissolve in 10 mL of ddH_2_O and aliquot into 1.5 mL tubes stored at -20 °C. Do not thaw and refreeze aliquots.

**14. 100 mM acetosyringone**

**Table:** 

Reagent	Final concentration	Quantity or volume
Acetylsyringone	100 mM	0.3924 g
Total	n/a	20 mL

Dissolve in 20 mL of DMSO and aliquot into 1.5 mL tubes stored at -20 °C. Do not thaw and refreeze aliquots.

**15. 50 mM biotin**

**Table:** 

Reagent	Final concentration	Quantity or volume
Biotin	50 mM	0.12 g
Total	n/a	10 mL

Dissolve in 10 mL of DMSO and aliquot into 1.5 mL tubes stored at -20 °C. Do not thaw and refreeze aliquots.

**16. 0.5 M MES**

**Table:** 

Reagent	Final concentration	Quantity or volume
MES	0.5 M	9.762 g
Total	n/a	100 mL

Use 10 N NaOH to adjust pH to 5.7. Store at RT.

**17. Infiltration buffer**

**Table:** 

Reagent	Final concentration	Quantity or volume
0.5 M MES (Recipe 16)	10 mM	2 mL
1 M MgCl_2 _(Recipe 9)	10 mM	1 mL
Total	n/a	100 mL

Prepare just before use. The solution is not stable if made in advance.

**18. Kanamycin 100 mg/mL**

**Table:** 

Reagent	Final concentration	Quantity or volume
Kanamycin	100 mg/mL	1 g
Total	n/a	10 mL

Weigh 1 g of kanamycin and dissolve in 9 mL of ddH_2_O in a 15 mL Falcon tube. Add ddH_2_O to a total volume of 10 mL, dissolve completely, pass through a 0.22 μm filter, and aliquot it. The stock may be kept at -20 °C for up to 1 year. Use 50 μg/mL as a working concentration.

**19. Spectinomycin 50 mg/mL**

**Table:** 

Reagent	Final concentration	Quantity or volume
Spectinomycin	50 mg/mL	0.5 g
Total	n/a	10 mL

Weigh 0.5 g of spectinomycin and dissolve in 9 mL of ddH_2_O in a 15 mL Falcon tube. Add ddH_2_O to a total volume of 10 mL, dissolve completely, pass through a 0.22 μm filter, and aliquot it. The stock may be kept at -20 °C for up to 1 year. Use 50 μg/mL as a working concentration.

**20. Rifampicin 25 mg/mL**

**Table:** 

Reagent	Final concentration	Quantity or volume
Rifampicin	25 mg/mL	0.25 g
Total	n/a	10 mL

Weigh 0.25 g of rifampicin and dissolve in 9 mL of DMSO in a 15 mL Falcon tube. Add DMSO to a total volume of 10 mL, dissolve completely, and aliquot it. The stock may be kept at -20 °C for up to 1 year. Use 50 μg/mL as a working concentration.

**21. 2 M glycine**

**Table:** 

Reagent	Final concentration	Quantity or volume
Glycine	2 M	150 g
Total	n/a	1,000 mL

Autoclave and store at RT.

**22. 20% (v/v) Triton X-100**

**Table:** 

Reagent	Final concentration	Quantity or volume
Triton X-100	20% (v/v)	10 mL
Total	n/a	50 mL

Store at RT.

**23. Extraction buffer (EB)**

**Table:** 

Reagent	Final concentration	Quantity or volume
1 M Tris-HCl pH 7.5 (Recipe 5)	50 mM	500 μL
5 M NaCl (Recipe 11)	150 mM	333.3 μL
1 M MgCl_2 _(Recipe 9)	2.5 mM	25 μL
50% glycerol (Recipe 8)	10% (v/v)	2 mL
20% Triton X-100 (Recipe 23)	0.5% (v/v)	250 μL
0.1 M PMSF	1 mM	100 μL
Protease inhibitor cocktail (100×)	1%	100 μL
RNase inhibitor	20 U/mL	5 μL
Total	n/a	10 mL

Prepare just before use. The solution is not stable if made in advance.

**24. Wash buffer**

**Table:** 

Reagent	Final concentration	Quantity or volume
1 M Tris-HCl pH 7.5 (Recipe 5)	20 mM	200 μL
5 M NaCl (Recipe 11)	150 mM	333.3 μL
1 M MgCl_2 _(Recipe 9)	2.5 mM	25 μL
50% glycerol (Recipe 8)	10% (v/v)	2 mL
20% Triton X-100 (Recipe 23)	0.2% (v/v)	100 μL
1 M DTT (Recipe 13)	0.5 mM	5 μL
Protease inhibitor cocktail (100×)	1%	10 μL
RNase inhibitor	20 U/mL	5 μL
Total	n/a	10 mL

Prepare just before use. The solution is not stable if made in advance.

**25. Dilution buffer**

**Table:** 

Reagent	Final concentration	Quantity or volume
1 M Tris-HCl pH 7.5 (Recipe 5)	20 mM	200 μL
5 M NaCl (Recipe 11)	150 mM	333.3 μL
1 M MgCl_2 _(Recipe 9)	2.5 mM	25 μL
50% glycerol (Recipe 8)	10% (v/v)	2 mL
Protease inhibitor cocktail (100×)	1%	100 μL
RNase inhibitor	20 U/mL	5 μL
Total	n/a	10 mL

Prepare just before use. The solution is not stable if made in advance.

**26. Elution buffer**

**Table:** 

Reagent	Final concentration	Quantity or volume
4× Laemmli buffer	2×	1 mL
50 mM biotin (Recipe 15)	5 mM	200 μL
10% (w/v) SDS (Recipe 29)	2% (w/v)	400 μL
1 M DTT (Recipe 13)	100 mM	200 μL
ddH_2_O	n/a	200 μL
Total	n/a	2 mL

Prepare just before use. The solution is not stable if made in advance.

**27. 10% (w/v) SDS**

**Table:** 

Reagent	Final concentration	Quantity or volume
SDS	10%	10 g
Total	n/a	100 mL

Store at RT.

**Caution:** SDS is a fine powder. It should be weighed under a fume hood to avoid inhalation or use a fume mask.

**28. 10% APS**

**Table:** 

Reagent	Final concentration	Quantity or volume
APS	10% (w/v)	1 g
Total	n/a	10 mL

Dissolve in 10 mL of ddH_2_O and aliquot into 1.5 mL tubes stored at -20 °C. Do not thaw and refreeze aliquots.

**29. 10× SDS running buffer**

**Table:** 

Reagent	Final concentration	Quantity or volume
Tris base	0.2501 M	30.3 g
Glycine	1.924 M	144.4 g
SDS	1% (w/v)	10 g
Total	n/a	1,000 mL

Prepare in advance and store at RT. Stable for 1 year.

**30. 1× SDS transfer buffer**

**Table:** 

Reagent	Final concentration	Quantity or volume
Tris base	0.02501 M	3.03 g
Glycine	0.1924 M	14.44 g
Methanol	20%	200 mL
Total	n/a	1,000 mL

Prepare just before use.

**31. 10× PBS**

**Table:** 

Reagent	Final concentration	Quantity or volume
NaCl	n/a	80.1 g
Na_2_HPO_4_	n/a	14.4 g
KCl	n/a	2 g
KH_2_PO_4_	n/a	2.7 g
Total	n/a	1,000 mL

Use 6 N HCl to adjust pH to 7.4. Autoclave and store at RT.

**32. 3% BSA blocking solution**

**Table:** 

Reagent	Final concentration	Quantity or volume
BSA	3% (w/v)	3 g
PBST	n/a	100 mL

Prepare just before use. The solution is not stable if made in advance.

**33. 0.1% (w/v) Ponceau staining solution**

**Table:** 

Reagent	Final concentration	Quantity or volume
Ponceau S	0.1% (w/v)	0.1 g
Glacial acetic	5% (w/v)	5 mL
Total	n/a	100 mL

Prepare in advance. The prepared Ponceau solution should be stored in the dark at 4 °C for one year.

**34. Protease K buffer**

**Table:** 

Reagent	Final concentration	Quantity or volume
1 M Tris-HCl pH 8.0 (Recipe 4)	30 mM	30 μL
Total	n/a	1 mL

Prepare just before use.

**35. Homogenization buffer**

**Table:** 

Reagent	Final concentration	Quantity or volume
1 M Tris-HCl pH 8.0 (Recipe 4)	100 mM	1,000 μL
5 M NaCl (Recipe 11)	100 mM	200 μL
0.5 M EDTA (Recipe 12)	0.5 mM	10 μL
1 M MgCl_2_ (Recipe 9)	2.5 mM	25 μL
0.1 M PMSF	1 mM	100 μL
*β-*mercaptoethanol	1%	100 μL
Total	n/a	10 mL

Prepare just before use. The solution is not stable if made in advance.

**36. 3 M NaAc**

**Table:** 

Reagent	Final concentration	Quantity or volume
NaAc	3 M	24.6102 g
Total	n/a	100 mL

Use 10 N NaOH to adjust pH to 5.2. Store at RT.

**Laboratory supplies**

*Note: Clean the laboratory supplies with RNaseZap^®^ RNase decontamination solution or use supplies indicated with DNase and RNase-free.*

1. Pipette set and tips (Eppendorf or similar)

2. Miracloth (Merck Millipore, catalog number: 475855)

3. Mesh (SefarNitex, catalog number: 03-100/44)

4. 1 mL disposable syringe

5. Mortar

6. Spatula

7. Gloves

8. 1.5 mL Protein LoBind microcentrifuge tubes (Eppendorf, catalog number: 022431081)

9. 15- or 50-mL polypropylene Falcon tubes or equivalent (DNase and RNase-free)

10. PCR tubes and caps (VWR, catalog number: 20170-010)

11. Syringe filter, 0.2 μm (Thermo Fisher Scientific, catalog number: 725-2520)

12. 1 mm gap sterile electroporation cuvette compatible with all common electroporators

13. Soil (Hasselfors P-soil or equivalent) mixed with sand (Rada sand) at a 5:1 (v/v) ratio: fill pots with the mix (e.g., pots of size 10 × 10 × 8 cm)

14. Reach-in or walk-in growth chambers at 22 °C during subjective daytime and 19 °C subjective night, with a cycle of 14/10 h light/dark (or 16/8 h light/dark). The light intensity at the leaf surface should be around 150 μE/m^2^/s from white light plant growth lamps. Pot watering schedule depends on the plant growth stage, avoiding flooding and extreme drought conditions (normally, 2–3 days/per time before flowering, 1–2 days/per time after flowering). Relative humidity can be kept constant and uniform at 50%–60%. For example, *Arabidopsis thaliana* and *Nicotiana benthamiana* plants can be grown in Aralab or Percival growth chambers.

## Equipment

*Note: Clean the laboratory equipment with RNaseZap^®^ RNase decontamination solution for all steps involving RNA.*

1. Vertical laminar flow clean bench

2. Incubator shaker that can reach 28 °C and 37 °C

3. Thermomixer (Eppendorf, model: 5382)

4. Ice bath

5. Microwave

6. Refrigerator (4 °C)

7. Freezer (-20 °C)

8. Ultra-low temperature freezer (-80 °C)

9. 37 °C and 42 °C water baths

10. Spectrophotometer

11. Ultraviolet gel imaging system (Azure Biosystems, model: c600)

12. NanoDrop Ultra UV-Vis spectrophotometer (Thermo Fisher Scientific, catalog number: NDULTRAGL)

13. Bio-Rad Gene Pulser X cell total electroporation system or similar

14. Table centrifuge (Eppendorf, model: centrifuge 5418, for RT)

15. Table centrifuge (Eppendorf, model: centrifuge 5417R, for 4 °C and -20 °C)

16. PCR machine (Bio-Rad, Veriti 96 wells, catalog number: 4375786)

17. Autoclave (Spire Integrated Solutions, model: PRIMUS)

18. Agarose gel electrophoresis equipment (Bio-Rad, model: PowerPac^TM^ Basic Power Supply, Horizontal Electrophoresis Systems)

19. Mini PROTEAN^®^ 3 System glass plates for casting in-house-made gels

20. Protein electrophoresis apparatus (e.g., Bio-Rad or equivalent)

21. Luminescent image analyzer

22. Odyssey infrared imaging system or similar

23. Real-time PCR system (e.g., LightCycler 480 System from Roche)

24. Vacuum pump (Techtum, model: MZ 2 NT)

25. Optional for sequencing: fluorimetric dsDNA quantification system (e.g., Qubit, Thermo Fisher)

26. Optional for sequencing: fragment analyzer (e.g., BioAnalyzer, Agilent)

27. Optional for sequencing: high-throughput sequencing device (e.g., Illumina) with corresponding library preparation kit

## Software and datasets

1. Construct and cloning design software (e.g., SnapGene 7.2 or similar)

2. R software (4.3.3) package with Bioconductor

3. Galaxy (https://usegalaxy.org)

4. Cytoscape v. 3.5.1 (https://cytoscape.org/)

## Procedure

**Overview and timeline**

The complete Turbo-RIP protocol consists of seven main phases:

A. Vector construction (1–2 weeks).

B. Plant transformation and selection (4–8 weeks for stable lines; 5 days for transient).

C. Validation of TurboID activity (2–3 days).

D. Sample preparation for RNA immunopurification (1–2 days).

E. Affinity purification and streptavidin capture (1–2 days).

F. RNA isolation (1–2 days).

G. cDNA library construction and RNA analysis (1–2 weeks).

Phases A–C have been detailed in our previous APEAL protocol [10,11].

**D. Sample preparation for RNA immunopurification**

**Figure 1.**
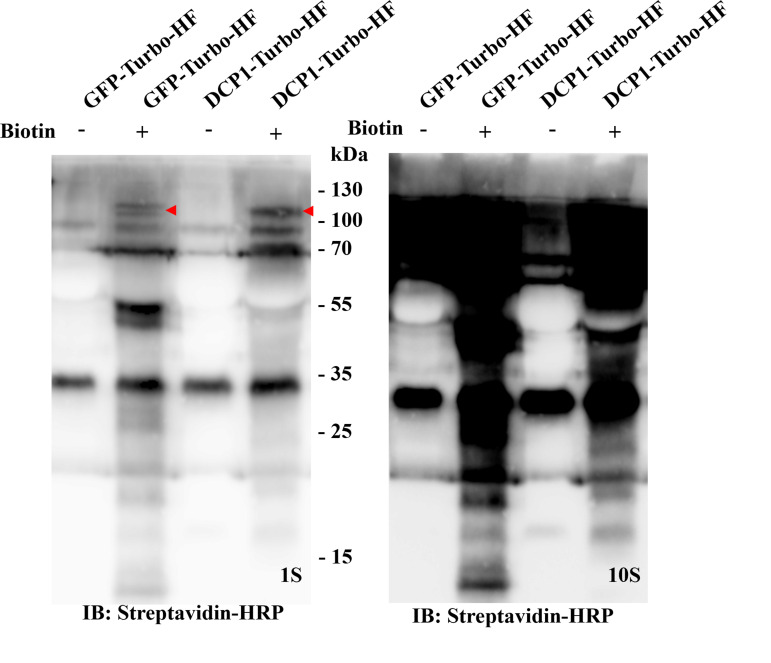
Sample preparation for the Turbo-RIP method. Five-day-old seedlings of a transgenic line of *p35S:GFP-TurboID-HF* (Control) and *p35S:DCP1-TurboID-HF* (Bait) were used for Turbo-RIP sample preparation. Perform 50 μM biotin treatment and 1% formaldehyde crosslinking steps according to the figure.

**Figure 2.**
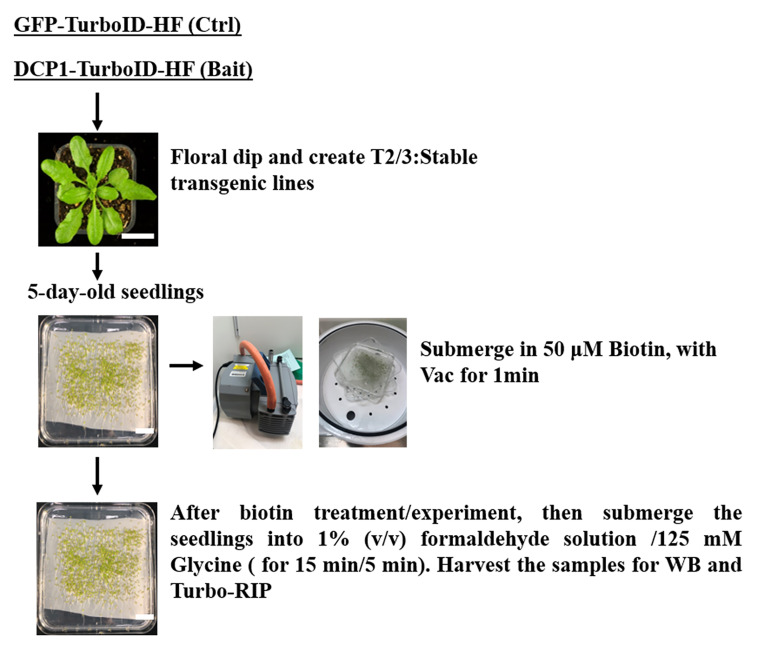
Western blot verification for the Turbo-RIP method in the *Nicotiana benthamiana* transient expression system. Transient expression of *p35S:GFP-TurboID-HF (Control, GFP-Turbo-HF)* and *p35S:DCP1-TurboID-HF(Bait, DCP1-Turbo-HF)* in healthy *N. benthamiana* plants (3–5 weeks) for two days, and then infiltrated with 50 μM biotin. After 24 h of biotin incubation, harvest the infiltrated leaf material for western blot. The immunoblots use Streptavidin-HRP (left side with 1 s exposure time, and right side with 10 s exposure time for clearer biotinylation smear). GFP-TurboID-HF and DCP1-TurboID-HF, marked by a red arrow.

1. Transgenic plant material preparation: Germinate the sterilized seeds from verified transgenics of T2 or T3 generation on 1/2 MS medium plates with mesh as shown in Figure 1. Place the plates vertically in a plant chamber (e.g., transgenic lines of *p35S:DCP1-TurboID-HF* and *p35S:GFP-TurboID-HF* were used in this protocol). Western blot verification of transient expression of the constructs in *N. benthamiana* as shown in Figure 2).

*Note: Cut meshes into 10 × 10 cm squares and sterilize by autoclaving before use. This makes it easy to remove the plants from the culture medium without causing damage to the roots.*

**Pause point:** Plant growth: associated time window up to the desired developmental stage of analysis (e.g., for seedlings, 5-day-old up to 2 weeks).

2. Biotin application: At the seedling stage (5-day-old *Arabidopsis* plants), prepare 50 μM biotin in infiltration buffer (add 50–75 mL for each plate to ensure the seedlings are submerged in the solution) and apply vacuum infiltration at 0.09 MPa for 1 min (Figure 1).

*Note: The infiltration step should be gentle to prevent damage to the seedlings.*

3. Pour out the extra biotin solution and put the plates back in the plant chamber for 24 h of biotin incubation.

*Note: Biotin incubation time needs to be tested for each experiment, as it may vary from case to case. We took 2-h intervals for the biotinylation test via WB and found that the 24-h incubation provided the best results.*

4. Heat stress treatment: Harvest the seedlings both for mock and 2-h heat stress treatment (37 °C, 2 h for our own experiment; DCP1 can form large condensates after a 2-h heat treatment. It can be easily adapted to other treatments or experiments). Alternatively, for older *Arabidopsis* plants, 50 μM biotin can be delivered to the mature leaves by biotin infiltration with a 1 mL syringe until full leaves are infiltrated.

**Figure 3.**
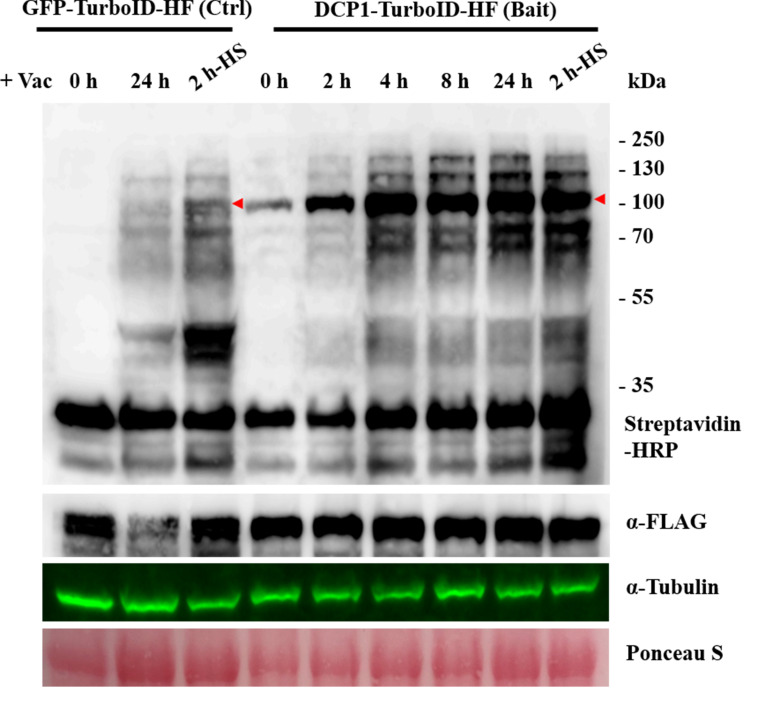
Western blot verification of transgenic lines for the TurboID activity. This figure presents examples of western blots used for stable *Arabidopsis* transgenic lines of *p35S:GFP-TurboID-HF* (Control) and *p35S:DCP1-TurboID-HF* (Bait) with biotin treatment. The western blots use Streptavidin-HRP and *α-*FLAG to detect biotinylation and bait expression, respectively. GFP-TurboID-HF and DCP1-TurboID-HF, marked by a red arrow. *α*-Tubulin and Ponceau S for the loading control. Note the presence of autobiotinylation of the baits. A band at approximately 35 kDa corresponds to an endogenous biotinylated protein. Ctrl denotes control. HF denotes His-FLAG tag.

**Caution:** A WB preliminary experiment can include time points of 0, 0.5, 1, 2, 4, 8, and 24 h post-infiltration with biotin (see Figure 3). Although earlier time points have not been tested in plants, successful results were obtained with a 10-min biotin incubation in non-plant systems (e.g., [8]). Users can change this according to tissue type or experimental goals.

5. After biotin application, collect around 2 g of fresh seedlings of the transgenic line into a 50 mL RNase-free tube with 36 mL of RNase-free ddH_2_O.

6. Add 1 mL of 37% (v/v) formaldehyde into the tube, mix well, and apply vacuum at 0.09 MPa for 15 min.

*Note: In case of difficult tissues, e.g., inflorescences, replace with 1–1.5 h and apply vacuum twice.*

**Caution:** Formaldehyde is carcinogenic; perform crosslinking and wash steps inside a chemical fume hood. The disposal of formaldehyde-containing waste needs to follow specific rules. Wear PPE requirements for all steps involving hazardous reagents. If the RNA concentration is extremely low, we suggest prolonging the formaldehyde crosslinking time or repeating the step.

7. Add 2.5 mL of 2 M glycine into the tube (125 mM glycine), mix well, and apply vacuum for 5 min to stop the crosslinking.

8. Stop the vacuum and rinse the seedlings to remove the formaldehyde with RNase-free ddH_2_O (50 mL/each time) around 5 times.

9. Material harvesting: Remove the RNase-free ddH_2_O completely with tissue paper. Flash‐freeze the seedlings in liquid N_2_.

**Pause point:** Carry on or store at -80 °C until further processing.

**E. Affinity purification and streptavidin capture**

1. Preparation of lysates: Mix powder of 5 mL pulverized *Arabidopsis* leaves or seedlings (~2 g fresh weight, FW) with 5 mL of extraction buffer at a 1:2 (w/v) ratio, or as determined experimentally in a 15 mL Falcon tube.

2. Protein extraction: Incubate plant extracts on a shaker at 4 °C for 15–30 min and then centrifuge at 13,000× *g* for 30 min at 4 °C to get a clear supernatant.

3. Capture of biotinylated proteins: Transfer the clarified supernatant to a new tube (pass through a two-layered Miracloth if necessary) and filter through PD‐10 columns to remove the extra free biotin according to the manufacturer's instructions.

**Critical:** The PD-10 step is extremely important. Use 15 mL Falcon tubes to catch the flowthrough to avoid sample loss (normally, 3.5 mL), remove extra free biotin from the supernatant, and ensure that streptavidin beads are not saturated. To prevent sample loss, add 25 mL of extraction buffer (without PI) to each PD-10 tube for equilibration.

4. Equilibrate Dynabeads^TM^ MyOne Streptavidin C1 in advance with 500 μL of cold wash buffer three times.

5. Incubate the biotin‐depleted flowthrough with 100 μL of Dynabeads^TM^ MyOne Streptavidin C1 at 4 °C for 2 h with gentle 360° rotation at 20–40 rpm.

*Note: Incubation can be done at 4 °C overnight if the bait is extremely low-expressed.*

**Critical:** Dynabeads^TM^ MyOne Streptavidin C1 beads have better affinity capability (≥ 2,800 pmol free biotin/mg, 1 μm diameter size for a large surface area per mg beads) than Dynabeads^TM^ M-280 Streptavidin beads (490–750 pmol free biotin/mg beads, 2.8 μm diameter size) (as used in the APEAL approach) for RNA immunopurification.

**Figure 4.**
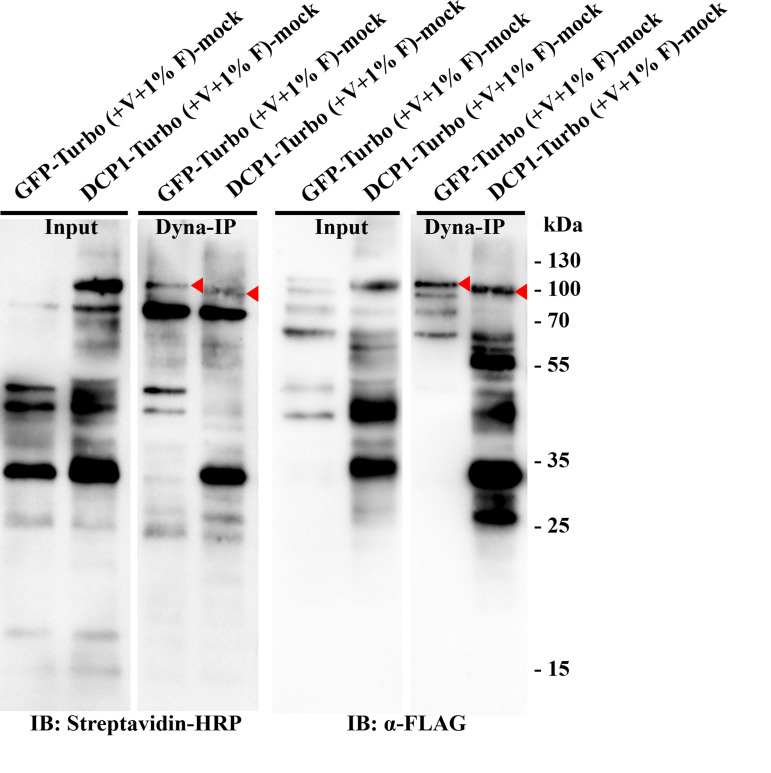
Western blot verification for the Turbo-RIP method. This figure presents examples of immunoblots used for verification of stable *Arabidopsis* transgenic lines of *p35S:GFP-TurboID-HF* (Control) and *p35S:DCP1-TurboID-HF* (Bait) with Dynabeads MyOne Streptavidin C1 immunoprecipitation. The western blots were incubated with Streptavidin-HRP and *α-*FLAG to detect biotinylation and bait expression, respectively. GFP-TurboID-HF and DCP1-TurboID-HF, marked by red arrows.

6. Clarification of biotinylated proteins from beads: Wash Dynabeads^TM^ MyOne Streptavidin C1 five times with 1 mL of wash buffer and twice with 1 mL of dilution buffer (no Triton X-100 or other detergent). Precipitate the beads using a DynaMag^TM^‐2 magnet.

7. Test capture efficiency of Dynabeads^TM^ MyOne Streptavidin C1 (optional): Use 5% beads for the test in the last wash step. Elute the proteins from the beads with 60 μL of 2× Laemmli buffer supplemented with 5 mM biotin, 2% (w/v) SDS, and 100 mM DTT and incubate at 95 °C for 20 min. The eluted samples can be used further for tests with immunoblots (see Figure 4).

**Critical:** Biotinylated proteins are tightly bound to streptavidin beads and may not elute easily with standard elution buffers. Adding 5 mM biotin to the elution buffer could help in the elution process.

**F. RNA isolation**

1. Resuspend the captured Dynabeads in 100 μL of protease K buffer with 2 μL of RNase inhibitor (0.8 U/μL) and 4 μL of proteinase K (0.8 μg/μL). Mix by pipetting gently and incubate at 55 °C for 30 min to elute protein and RNAs from the beads.

2. Extract the RNA: Transfer the solution into a new 1.5 mL low-binding tube, add homogenization buffer to make up the volume to 400 μL, and mix vigorously.

**Caution:** Homogenization buffer (w/o *β-*mercaptoethanol) can be prepared in advance and kept at room temperature. *β-*mercaptoethanol must be added prior to use in a fume hood.

3. Add 400 μL of phenol-chloroform-isoamyl alcohol (pH 4–5), mix well, and centrifuge at max speed (> 19,000× *g*) for 15 min at 4 °C.

*Note: Conduct the experiment inside a chemical fume hood, as the phenol-chloroform-isoamyl alcohol mixture is hazardous.*

4. Transfer the upper phase (300–400 μL) to a new tube and add an equal volume of isopropanol, with 0.1 volumes of 3 M NaAc, pH 5.2, and 2 μL of GlycoBlue. Incubate at -20 °C for 1–2 h.

**Critical:** Handling of the organic phase is important; take the upper phase only. Note that phase-lock tubes can improve reproducibility.

5. Centrifuge at max speed (> 19,000× *g*) for 20 min at 4 °C.

**Critical:** GlycoBlue is used for better visualization of precipitated RNA and should correspond to a tiny blue pellet visible at the bottom of the tube.

6. Rinse the pellet carefully with 750 μL of 75% ethanol, mix by two inversions, centrifuge at max speed (> 19,000× *g*) for 5 min at 4 °C, and remove the supernatant completely.

**Critical:** Take care not to disturb the RNA pellet, and do not lose the blue pellet when removing the ethanol solution. It may cause yield variability.

7. Repeat step F6.

8. Air-dry the pellet in the hood.

**Critical:** Clean the hood in advance with RNaseZap^®^ RNase decontamination solution to keep the environment as clean as possible.

9. Resuspend the pellet in 15 μL of DNase-, RNase-, and protease-free water.

**Pause point:** Carry on or store at -80 °C until further processing.

**G. cDNA library construction and RNA analysis**

1. Treat the RNAs (initial around 2 μg) with DNase I and rRNA removal using the RiboMinus^TM^ Kit following the manufacturer’s protocol (we normally obtain around 1 µg of RNA after DNase I and rRNA removal). Proceed immediately to the next step. Use Qubit RNA HS Assay Kits to measure RNA concentration.

2. Sequencing: Prepare cDNA libraries with NEBNext Ultra II RNA Library Prep with sample purification beads and NEBNext Multiplex Oligos for Illumina (Dual Index Primers Set 1).

**Figure 5.**
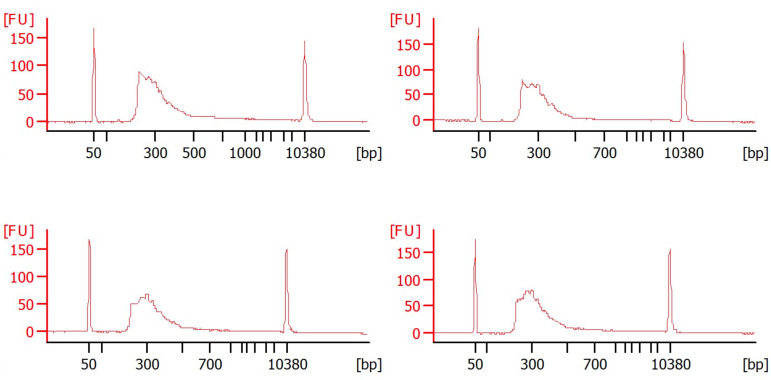
cDNA library preparation. Four Bioanalyzer electropherograms, each plotting the size and concentration of a different DNA sample. The x-axis represents DNA fragment size in base pairs (bp), ranging from approximately 50 to over 10,000 bp. The y-axis shows the amount of DNA in fluorescence units (FU). Each plot shows a lower marker (~50 bp) and an upper marker (~10,380 bp), which are used as internal standards for accurate sizing and quantification. The broad smear or peak in the center of each plot, generally between 100 and 800 bp, represents the DNA library, prepared for next-generation sequencing. The shape and location of this smear indicate the size distribution of the DNA fragments. The presence of sharp, small peaks, particularly those below 100 bp, might indicate undesirable side products like primer dimers or adaptor dimers. The overall shape and peak height of each electropherogram can be used to assess the quality, quantity, and size distribution of the DNA libraries.

**Critical:** Quantify the samples precisely using a fluorimetric dsDNA quantification system (such as the Qubit) and analyze fragment sizes precisely using a microcapillary electrophoresis trace analyzer (such as the Agilent BioAnalyzer; see Figure 5). After size selection and quantification, prepare the library with a kit (usually, an Illumina Sequencing kit). Because of the low RNA/cDNA concentrations, it is essential to work in a very clean environment.

3. Synthesize cDNA with a reverse transcriptase (RT). We use, for example, SuperScript IV Reverse transcriptase following the manufacturer’s protocol, but several alternatives are available.

**Critical:** Make sure that you use a reverse transcriptase with high performance. For each sample, run a “no reverse transcription” control (no Super-Script IV added) and two technical replicates.

4. Use approximately 500 ng of the RNA for the reverse transcription (RT). Add 0.5 μL of 100 μM oligo d(T) or 1.0 μL of 50 μM random hexamers or specific primers, 1 μL of 10 mM dNTP, and complete the volume up to 13 μL with water.

5. Incubate the RNA mix at 65 °C for 5 min and transfer immediately to ice for at least 1 min.

6. In a separate tube, prepare the RT mix by adding 4 μL of 5× SSIV buffer, 1 μL of 0.1 M DTT, 1 μL of 40 U/μL RNase inhibitor, 0.5 μL of SuperScript IV RT, and 0.5 μL of water. Mix by slowly pipetting up and down.

7. Add the RT mix to the RNA mix and incubate at 55 °C for 15 min.

8. Inactivate the reaction by heating at 80 °C for 10 min.

**Pause point:** Store the cDNA at -80 °C or use it immediately for qPCR analysis.

9. qRT-PCR quantification: Quantify the immunoprecipitated RNA with qRT-PCR to examine the enrichment of the RNAs of interest. Design the primers to amplify the sequences of interest and several controls. Analyze the raw data from the qPCR using the 2^-ΔΔCT^ analysis method.

10. Sequence DNA libraries with a paired-end sequencing strategy to produce 2× 150-bp reads using Novogene sequencers (Novogene, England).

**Critical:** When total RNA from the same plant extracts is used as a control for specific RNA enrichment comparison, take a certain number of plants for RNA extraction before the crosslinking step or the de-crosslinking step is needed.

11. Perform Gene Ontology term (GO) analysis on hits using the Panther database or BioConductor package in R, as detailed in [13].

12. Use Cytoscape 3.5.1 for visualization. Upload tab-delimited files containing input data. The default layout is an edge-weighted spring-embedded layout, with the NormSpec used as edge weight. Rearrange the nodes manually from this layout to increase visibility and highlight specific proximity interactions. Export the layout as a PDF and convert to a TIFF file.

## Data analysis

**Overview of analysis workflow**

The Turbo-RIP data analysis consists of four main components: (1) validation of TurboID activity through western blot analysis; (2) quality assessment of isolated RNA, (3) qRT-PCR analysis for targeted validation, and (4) comprehensive RNA-seq data processing and interpretation. Each component requires specific quality control metrics and statistical approaches to ensure reproducible and biologically meaningful results. This comprehensive analytical framework ensures that Turbo-RIP data yield biologically meaningful insights into RNA landscapes within plant biomolecular condensates.

**A. Validation of TurboID activity: Western blot quantification**

1. Image acquisition and processing

a. Acquire chemiluminescent images using a Luminescent Image Analyzer or similar system.

b. For quantitative analysis, ensure images are not oversaturated (no pixel intensity values at maximum).

c. Save images in TIFF format at 16-bit depth for optimal dynamic range.

d. Use consistent exposure times across all samples within an experiment.

2. Densitometry analysis

a. Perform densitometry using ImageJ (Fiji distribution, version 1.53 or later) or similar software (see, e.g., https://imagej.net/software/fiji/downloads).

b. For each lane, draw rectangular regions of interest (ROIs) of equal size encompassing the entire band.

c. Subtract background using the “rolling ball” algorithm (radius = 50 pixels).

d. Normalize biotinylation signals to loading controls (tubulin or Ponceau S total protein staining).

3. Quality criteria for TurboID activity

a. Autobiotinylation: TurboID-fusion protein should show a clear biotinylation signal at the expected molecular weight.

b. Signal-to-noise ratio: Biotinylation signal in + biotin samples should be ≥ 2-fold higher than in no-biotin controls.

c. Time-course validation: For preliminary experiments, biotinylation may increase progressively over time (0, 0.5, 1, 2, 4, 8, 24 h).

d. Expression levels: Bait protein expression should be detectable by anti-FLAG (or equivalent tag antibody) if they are tagged.

e. Endogenous control: The ~35 kDa endogenous biotinylated protein band may be consistent across all samples.

4. Expected results: Figure 3 shows representative western blots demonstrating successful TurboID activity with:

a. Clear autobiotinylation of TurboID-fusion proteins (marked by red arrows).

b. Minimal background in no-biotin controls.

c. Consistent expression levels across transgenic lines.

**B. RNA quality assessment: Initial RNA quantification and purity**

1. Concentration measurement

a. Use NanoDrop spectrophotometer for initial assessment.

b. Expected yield: 50–500 ng of total RNA from 2 g of starting tissue.

c. If the yield is < 50 ng, consider scaling up the starting material or see General notes and troubleshooting section.

2. Purity assessment

a. Measure A_260/A280_ ratio: should be 1.8–2.0 for pure RNA.

b. Measure A_260/A230_ ratio: should be > 1.8 (values < 1.5 indicate salt or phenol contamination).

c. If purity is low, perform additional phenol–chloroform extraction and ethanol precipitation.

3. RNA integrity assessment

a. Use Agilent Bioanalyzer 2100 with RNA Nano or Pico chips.

b. Calculate RNA integrity number (RIN): acceptable values are RIN ≥ 7.0.

c. For degraded samples (RIN < 7), troubleshoot RNase contamination before proceeding.

d. Expected profile: clear 18S and 28S ribosomal peaks (before rRNA depletion) with minimal baseline elevation.

4. Post-rRNA depletion quality control

a. After RiboMinus treatment, rRNA content should be < 10% of total reads.

b. Use Qubit RNA HS Assay kit for accurate quantification of low-concentration samples.

c. Expected yield after DNase I and rRNA removal: ~1 μg from initial 2 μg input.

**C. Quantitative RT-PCR analysis**

**C1. Experimental design**

1. Sample organization

a. Include biological replicates: minimum 2, preferably 3 independent biological replicates.

b. Technical replicates: 2–3 technical replicates per biological sample.

c. Controls:

• No-RT controls (no reverse transcriptase) to assess genomic DNA contamination.

• Biotin controls (samples without biotin treatment).

• GFP-TurboID or empty vector controls (nonspecific capture).

2. Reference gene selection

a. Use at least two reference genes for normalization.

b. Recommended reference genes for *Arabidopsis: UBQ10* (AT4G05320), *PP2A* (AT1G13320), or *ACT2* (AT3G18780).

c. Verify reference gene stability across experimental conditions using geNorm or NormFinder algorithms.

**C2. qRT-PCR data analysis**

1. Quality control of raw data

a. Inspect amplification curves: Should show exponential phase, linear phase, and plateau.

b. Check melting curves: A single sharp peak indicates specific amplification.

c. Reject wells with Ct values > 35, abnormal amplification curves, multiple melting peaks, or technical replicate variation > 0.5 Ct.

2. Calculation of enrichment

a. Use the 2^-ΔΔCt^ method for relative quantification:

b. * ΔCt = Ct (target gene) - Ct (reference gene)

c. * ΔΔCt = ΔCt (IP sample) – ΔCt (control)

d. * Fold enrichment = 2^-ΔΔCt^

e. Alternative method for absolute quantification: standard curve method.

f. Generate standard curves using serial dilutions (10^2^ to 10^7^ copies).

g. Calculate copy numbers based on Ct values.

h. Report as copies per ng input RNA.

3. Statistical analysis

a. Calculate mean and standard error (SEM) or standard deviation (SD) from biological replicates.

b. Perform statistical testing: Two-group comparison, Student's t-test (normally distributed data) or Mann–Whitney U test (non-parametric); multiple comparisons, one-way ANOVA with post-hoc Tukey's HSD test. Significance threshold: P < 0.05.

c. Software: GraphPad Prism (version 9 or later), R (version 4.0 or later), or equivalent.

4. Interpretation guidelines

a. Significant enrichment: Fold change ≥ 2.0 with P < 0.05 compared to control.

b. Expected positive controls: Known condensate-associated RNAs should show ≥ 5-fold enrichment.

c. Expected negative controls: Housekeeping genes not associated with condensates should show < 1.5-fold enrichment.

d. Specificity: Bait-specific targets should show higher enrichment in bait samples vs. GFP-TurboID controls.

**D. RNA-seq data processing and analysis**

**D1. Raw data quality control**

1. Initial quality assessment

a. Use FastQC (version 0.11.9 or later) to assess raw sequencing reads.

b. Key metrics to evaluate:

• Per base sequence quality: Phred scores should be > 30 for > 90% of bases.

• Per sequence quality scores: Median Phred score > 28.

• Sequence length distribution: Should match expected library size (typically 150 bp).

• GC content: Should approximate the expected distribution for *Arabidopsis* (~36%).

• Adapter content: Should be minimal (< 5%).

• Duplication levels: High duplication is expected for RNA-seq, but excessive duplication (> 50%) may indicate PCR artifacts.

2. Read preprocessing

a. Trim adapters and low-quality bases using Trimmomatic (version 0.39 or later) or Trim Galore! (version 0.6.7 or later).

b. Parameters for Trimmomatic:

c. ILLUMINACLIP: TruSeq3-PE.fa:2:30:10

d. LEADING: 3

e. TRAILING: 3

f. SLIDINGWINDOW: 4:15

g. MINLEN: 36

h. Discard reads < 36 bp after trimming.

i. Re-run FastQC on trimmed reads to verify improvement.

**D2. Read alignment and quantification**

1. Reference genome preparation

a. For *Arabidopsis thaliana*: Use TAIR10 genome assembly.

b. Download from https://www.arabidopsis.org/ or Ensembl Plants.

c. Build genome index using chosen aligner (STAR or HISAT2).

2. Alignment Using STAR

a. Software: STAR aligner (version 2.7.10a or later).

b. Index generation (for code, see File S1).

3. Alternative alignment using HISAT2

a. Software: HISAT2 (version 2.2.1 or later).

b. Index generation (for code, see File S1).

4. Quality control of aligned reads

a. Use Qualimap (version 2.2.1) or RSeQC (version 4.0.0) for post-alignment QC.

b. Key metrics:

• Uniquely mapped reads: Should be > 70%.

• Multi-mapped reads: Typically, 10%–20% for plant genomes.

• Unmapped reads: Should be < 10%.

• Insert size distribution: Median ~300–500 bp for typical library prep.

• Gene body coverage: Should show relatively uniform coverage (5′ to 3′ bias < 3:1).

• rRNA content: Should be < 10% after RiboMinus treatment.

5. Gene-level quantification

a. Use featureCounts (Subread package version 2.0.3 or later) for read counting.

b. Command (for code, see File S1).

c. Parameters:

• T: number of threads.

• p: count fragments (paired-end mode).

• B: only count read pairs with both ends aligned.

• C: do not count chimeric fragments.

d. Alternatively, use HTSeq-count or STAR's built-in quantification (--quantMode GeneCounts).

**D3. Normalization and differential expression analysis**

1. Data import and filtering

a. Import count matrix into R (version 4.3 or later).

b. Load required packages (see File S1).

2. Normalization methods

a. Option A: DESeq2 (recommended for most applications; for R code, see File S1):

• Run DESeq2 normalization and differential expression.

• Extract normalized counts.

• normalized_counts <- counts (dds, normalized=TRUE).

• Get results for Bait vs. Control comparison.

b. Option B: edgeR (Alternative method):

• Create DGEList object.

• Calculate normalization factors (TMM method).

• Estimate dispersions.

• Differential expression analysis.

3. Quality control visualizations

a. Sample clustering and correlation (for code, see File S1):

• Variance stabilizing transformation for visualization.

• Sample distance heatmap.

• Pearson correlation heatmap.

b. Expected results: Biological replicates should cluster together with Pearson r > 0.8.

c. Interpretation: “+biotin” samples should cluster separately from “-biotin” controls.

4. Principal component analysis (PCA) (for code, see File S1)

a. Perform PCA.

b. Plot PCA.

c. Quality threshold: PC1 should explain > 50% of variance, separating +biotin from -biotin samples.

d. Expected results: Distinct clustering of bait vs. control samples, with biological replicates grouping together.

**D4. Identification of enriched RNAs** (for code, see File S1)

a. Significance thresholds.

b. Define significance criteria.

c. Extract significantly enriched genes.

**D5. Visualization of results**

1. MA plot

a. Shows the relationship between expression level (x-axis) and fold change (y-axis).

b. Significantly enriched genes highlighted in red.

2. Volcano plot: Create a volcano plot (for code, see File S1).

3. Heatmap of top enriched genes

a. Select the top 50 enriched genes.

b. Create heatmap (for code, see File S1).

4. Functional enrichment analysis (for code, see File S1)

a. Gene ontology (GO) enrichment analysis.

b. Using clusterProfiler in R.

c. Convert gene IDs to the appropriate format.

d. GO enrichment analysis.

e. Visualize results.

f. Perform for all GO categories.

g. Using Panther Classification System.

h. Upload gene list to Panther website (http://pantherdb.org/).

i. Select *Statistical overrepresentation test*.

j. Choose organism: *Arabidopsis thaliana*.

k. Select annotation dataset: GO biological process complete.

l. Test type: Fisher's Exact with FDR correction.

m. Significance threshold: FDR < 0.05.

n. Expected results for processing body analysis: Enriched GO terms should include:

• mRNA catabolic process (GO:0006402).

• RNA-mediated gene silencing (GO:0016246).

• Deadenylation-dependent decapping of nuclear-transcribed mRNA (GO:0000290).

• Cytoplasmic stress granule assembly (GO:0034063).

5. KEGG pathway enrichment (for code, see File S1)

a. KEGG enrichment analysis.

b. Visualize pathways.

6. Network visualization with Cytoscape

a. Preparing input files (for code, see File S1).

b. Create node table (gene attributes).

c. Create edge table (gene–gene interactions).

d. If using protein–protein interaction data or co-expression network.

e. Cytoscape Workflow

i. Open Cytoscape (version 3.9 or later).

ii. Import network: *File* → *Import* → *Network from File* (select edges.txt).

iii. Import node attributes: *File* → *Import* → *Table from File* (select nodes.txt).

iv. Set visual style:

• Node color: Map to log_2_FC (red for enriched, blue for depleted).

• Node size: Map to baseMean (expression level).

• Edge width: Map to correlation coefficient.

v. Apply layout: *Layout* → Edge-weighted spring embedded.

• Use correlation as edge weight.

vi. Export: *File* → *Export* → *Network to Image* (save as PDF or TIFF).

f. Expected network properties

• Hub genes (highly connected): Should be central to condensate function.

• Modules: Expect clustering of functionally related genes.

• Network diameter: Typical values 4–6 for biological networks.

7. Comparison with control datasets

a. Total RNA control analysis.

• Create a design with three conditions: Bait+Biotin, Control+Biotin, and total RNA (if total RNA from the same plant extracts is used as input control) (for code, see File S1).

• Compare Bait vs. total RNA (specific enrichment).

b. Specificity analysis:

• Identify bait-specific transcripts.

• Calculate enrichment ratio.

• Expected: enrichment_ratio > 2 indicates high specificity.

8. Reproducibility assessment

a. Biological replicate concordance:

• Calculate correlation between replicates (for code, see File S1).

• Expected: Pearson r > 0.8 (excellent), 0.7–0.8 (good), < 0.7 (troubleshoot).

• Scatter plot (for code, see File S1).

b. Technical validation:

• Compare qRT-PCR results with RNA-seq fold changes.

• Calculate Spearman correlation: should be r > 0.7.

**E. Interpretation guidelines and expected results**

Overall experimental success criteria: A successful Turbo-RIP experiment should meet the following benchmarks:

1. Western blot validation

a. Clear TurboID activity (autobiotinylation visible).

b. Signal-to-noise ratio ≥ 5:1 (+biotin vs. -biotin).

c. Consistent bait expression across replicates.

2. RNA quality

a. RNA yield: 50–500 ng from 2 g of tissue.

b. RIN score ≥ 7.0.

c. rRNA content < 10% after depletion.

d. A_260/A280_ ratio: 1.8–2.0.

3. Library quality

a. Fragment size distribution: 200–800 bp peak.

b. Adapter dimer content: < 5%.

c. Sequencing depth: > 20 million paired-end reads per sample.

d. Uniquely mapped reads: > 70%.

4. Biological reproducibility

a. Pearson correlation between replicates: r > 0.8.

b. PC1 explains > 50% variance (PCA).

c. Distinct clustering of experimental groups.

5. Enrichment metrics

a. Number of enriched transcripts: 500–2,000 (dependent on bait).

b. Known positive controls: ≥ 5-fold enrichment.

c. Specificity: Bait-specific enrichment over GFP-TurboID control.

**F. Biological interpretation**

1. Strong confidence targets (high priority for follow-up):

a. Log_2_FC ≥ 2 (4-fold enrichment).

b. Adjusted p-value < 0.01.

c. Consistent enrichment across biological replicates.

d. Validation by qRT-PCR.

2. Moderate confidence targets (candidates for further validation):

a. Log_2_FC 1-2 (2–4-fold enrichment).

b. Adjusted p-value < 0.05.

c. May require additional experimental validation.

3. Context-dependent targets:

a. Some RNAs may show condition-specific association (e.g., stress-induced).

b. Compare results across different treatments or timepoints.

**G. Data deposition**

Deposit raw and processed sequencing data in public repositories:

• Gene Expression Omnibus (GEO): https://www.ncbi.nlm.nih.gov/geo/

• ArrayExpress: https://www.ebi.ac.uk/arrayexpress/

The RNA sequencing data of the Turbo-RIP and total RNA datasets have been submitted to the BioStudies database [15] under the accession number S-BSST1096 and can be accessed through the following link: https://www.ebi.ac.uk/biostudies/studies/S-BSST1096?key=58afa48a-c6c2-4366-b059-d2504392a2b2. Python scripts may be retrieved from https://github.com/Nwntastz/RNA_proxitome_paper.

**Conclusions**

Turbo-RIP provides a robust, reproducible method for capturing condensate-associated RNAs in plants. The protocol combines the spatial resolution of proximity labeling with the stability of crosslinking, enabling comprehensive analysis of dynamic RNA-protein assemblies. With standard molecular biology equipment and reagents, any plant laboratory can implement Turbo-RIP to study biomolecular condensates.

## Validation of protocol

This protocol has been used and validated in the following research articles:

- Liu et al. [13] A proxitome-RNA-capture approach reveals that processing bodies repress coregulated hub genes. *Plant Cell*. 2024 Feb 26;36(3): 559–584. doi: 10.1093/plcell/koad288.
- Liu et al. [14]. An actin remodeling role for *Arabidopsis* processing bodies revealed by their proximity interactome. *EMBO J*. 2023 May 2;42(10): e111885. doi: 10.15252/embj.2022111885.

## General notes and troubleshooting

**General notes**

**Critical parameters**

1. Temperature: TurboID functions optimally at 22–25 °C. Higher temperatures may increase nonspecific labeling.

2. Biotin concentration: 50 μM is sufficient for most applications but can be increased to 100 μM for low-expressing proteins.

3. Crosslinking time: 15 min is optimal for leaf tissue. Dense tissues may require longer.

4. Bead amount: 100 μL of beads can capture biotinylated proteins from up to 5 mg of total protein.

5. To avoid RNA degradation, use RNaseZap^®^ RNase decontamination solution to clean the equipment and tables. Prepare all buffers with DEPC-treated water.

**Method limitations**

1. Requires genetic transformation.

2. May not capture very transient interactions (< 10 min).

3. Background from endogenous biotinylated proteins.

4. Formaldehyde crosslinking may introduce bias.

**Applications and extensions**

Turbo-RIP can be adapted for:

1. Stress-responsive condensate analysis.

2. Developmental stage-specific RNA capture.

3. Comparative analysis across mutant backgrounds.

4. Integration with proteomics (parallel protein analysis).

**Troubleshooting**

**Table:** 

Problem	Possible cause	Solution
Low biotinylation signal	Insufficient biotin uptake	Increase biotin concentration to 100 μM; extend incubation to 24 h.
	Inactive TurboID	Verify construct sequence; test enzyme with purified protein.
	Low expression	Use stronger promoter; select high-expressing lines.
High background	Endogenous biotinylation	Include more washing steps; use no-biotin controls.
	Nonspecific binding	Reduce bead amount; increase 300 mM salt in wash buffer.
RNA degradation	RNase contamination	Use DEPC-treated water; add more RNase inhibitor; clean all materials with NaOH or another RNase-containing buffer.
	Over-crosslinking	Reduce formaldehyde time to 10 min.
Low RNA yield	Insufficient starting material	Scale up to 5 g of tissue.
	Poor lysis	Optimize tissue grinding; increase the extraction buffer.

## Supplementary information

The following supporting information can be downloaded here:

1. File S1. Code for data analysis
